# 3D Printing Technologies in Personalized Medicine, Nanomedicines, and Biopharmaceuticals

**DOI:** 10.3390/pharmaceutics15020313

**Published:** 2023-01-17

**Authors:** Dolores R. Serrano, Aytug Kara, Iván Yuste, Francis C. Luciano, Baris Ongoren, Brayan J. Anaya, Gracia Molina, Laura Diez, Bianca I. Ramirez, Irving O. Ramirez, Sergio A. Sánchez-Guirales, Raquel Fernández-García, Liliana Bautista, Helga K. Ruiz, Aikaterini Lalatsa

**Affiliations:** 1 Department of Pharmaceutics and Food Science, School of Pharmacy, Complutense University of Madrid, 28040 Madrid, Spain; 2 Instituto Universitario de Farmacia Industrial, Universidad Complutense de Madrid, 28040 Madrid, Spain; 3 Department of Physical Chemistry, Complutense University of Madrid, 28040 Madrid, Spain; 4 Institute of Pharmacy and Biomedical Sciences, University of Strathclyde, 161 Cathedral Street, Glasgow G4 0RE, UK; 5 CRUK Formulation Unit, School of Pharmacy and Biomedical Sciences, University of Strathclyde, 161 Cathedral Street, Glasgow G4 0RE, UK

**Keywords:** personalized medicines, 3D printing, FDM, fuse deposition modelling, SLA, stereolithography, PAM, pressure-assisted microsyringes, SLS, selective laser sintering, bioprinting, nanomedicines, nanoparticle, peptide hydrogel, microfluidic chip

## Abstract

3D printing technologies enable medicine customization adapted to patients’ needs. There are several 3D printing techniques available, but majority of dosage forms and medical devices are printed using nozzle-based extrusion, laser-writing systems, and powder binder jetting. 3D printing has been demonstrated for a broad range of applications in development and targeting solid, semi-solid, and locally applied or implanted medicines. 3D-printed solid dosage forms allow the combination of one or more drugs within the same solid dosage form to improve patient compliance, facilitate deglutition, tailor the release profile, or fabricate new medicines for which no dosage form is available. Sustained-release 3D-printed implants, stents, and medical devices have been used mainly for joint replacement therapies, medical prostheses, and cardiovascular applications. Locally applied medicines, such as wound dressing, microneedles, and medicated contact lenses, have also been manufactured using 3D printing techniques. The challenge is to select the 3D printing technique most suitable for each application and the type of pharmaceutical ink that should be developed that possesses the required physicochemical and biological performance. The integration of biopharmaceuticals and nanotechnology-based drugs along with 3D printing (“nanoprinting”) brings printed personalized nanomedicines within the most innovative perspectives for the coming years. Continuous manufacturing through the use of 3D-printed microfluidic chips facilitates their translation into clinical practice.

## 1. Personalised Medicine

The Horizon 2020 Advisory Group has defined personalised medicine as “a medical model using characterization of individuals’ phenotypes and genotypes such as lifestyle data, medical imaging or molecular profiling, for tailoring the right therapeutic strategy for the right person at the right time, and/or to determine the predisposition to disease and/or to deliver timely and targeted prevention” [1,2]. According to the Research and Innovation Unit of the European Commission, personalized medicines address the challenges of conventional medicines that are not effective in treating a large number of patients and rising healthcare costs due to more prevalent chronic disease and an ageing population. In this context, personalized medicines allows for custom-made prevention and treatment strategies for individuals or groups of patients that are optimised for them with no money wasted on trial-and-error treatments [1]. 

Apart from personalized medicine, other terms are also used to describe the same concept, such as individualized medicine, precision medicine, stratified medicine, pharmacogenomics, genomic medicine, and P4 medicines, including personalized, predictive, preventive, and participatory [3]. 3D printing technologies have emerged as a powerful tool for manufacturing personalized medicines, providing healthcare professionals with a huge arsenal of different techniques to fabricate custom-made medicines and medical devices. Initially, 3D printing technologies were developed to produce tablets moving from simple formulations, just containing the drug in a specific dose not commercially available, to complex systems, containing all drugs required and combining different release profiles within the same tablet adapted to the patient’s need. 3D printing also has enabled the manufacturing of personalized metallic prostheses and parenteral implants and other types of medical devices. In the last years, the application of 3D printing technologies in the manufacturing of medicines containing biopharmaceuticals or drugs encapsulated within nanovehicles, known as nanomedicines, is attracting more and more attention in the scientific community. In this review, the different 3D printing technologies commonly used in the development and fabrication of personalized medicines will be covered, such as material extrusion techniques (fused deposition modelling (FDM) and semisolid extrusion (SSE)) and vat photopolymerisation (stereolithography, SLA). 3D-printed medicines containing biopharmaceuticals and nanomedicines will also be discussed. 

## 2. 3D Printing of Medicines

3D printing involves the accumulation of a series of 2D layers that, as a result, give rise to a 3D geometry. The versatility of 3D printing techniques together with the lower cost of the necessary equipment means that these techniques are becoming increasingly popular, which has enabled the maturation of the technologies for translation into clinical practice. Applying 3D printing technologies allows pharmacological therapies to be personalized in an extremely precise and individualized way adapted to the needs of each patient.

### 2.1. What Applications Can 3D Printing Have for Healthcare Professionals?

**Figure 1:**
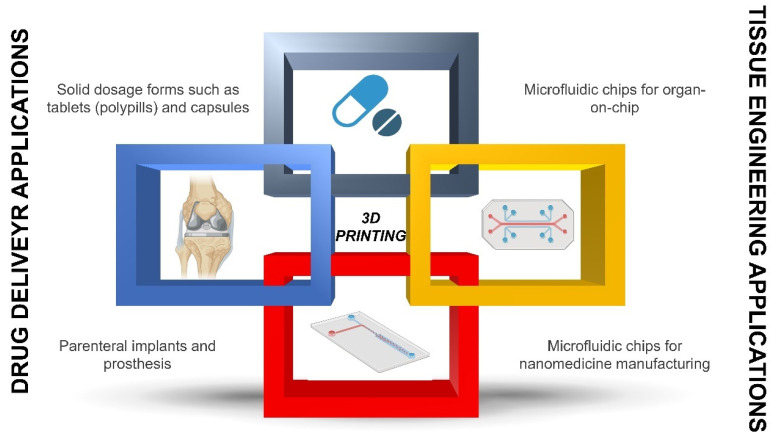
Application of 3D printing in personalized medicine.

3D printing can have endless possibilities from the 3D printing of medicines (topical, oral, and parenteral dosage forms) to tissue engineering and microfluidic organ-on-chips (Figure 1). 3D-printed dosage forms, such as tablets or capsules, are of increased interest, considering the licensing of the first 3D-printed product (Spritam©), while the development of personalized implants adapted to the dimensions of the cavity or tissue of interest for each patient is also very popular [4,5,6,7]. Implants are typically prepared from 3D-printed hydrogels that exhibit high water content within their structure while they remain biocompatible and biodegradable, while prostheses are produced with metal printers according to computerized images obtained with imaging techniques, such as MRI or axial tomography. 4D printing, with time being the fourth dimension, is under research as when implants are integrated into the patient’s body and can change shape over time or when they are integrated into patients’ body, such as breast implants after a mastectomy [8,9]. 

Within tissue engineering applications, 3D printing is mainly used at two levels. The first one is regarding the 3D printing of scaffolds for cell cultures. Conventional 2D cultures have many limitations, and although useful for initial screening, the data on efficacy and toxicity are far from in vivo data. However, 3D printing techniques, especially bioimprinting, can create 3D cell cultures and thus 3D models, also known, as organ-on-a-chip, that mimic human tissue much more closely and thus can mimic human response [10,11]. For example, the hepatic metabolism of drugs can be evaluated by printing well-defined hepatocyte architectures for testing, or similarly, the renal clearance can be reproduced by printing nephronlike structures [12]. Connecting these organ-on-chips in series can allow models to understand the overall permeability or clearance mimicking human data [13,14]. This allows the reduction of animals used in experimentation since these chips are much more similar to the human body, and more reliable results can be obtained with in vitro tools before moving forward to animal preclinical studies. Using 3D printers, not only the 3D tissue architecture can be printed but also the chip itself. 3D printing of microfluidic chips has been recently demonstrated [15,16,17,18]. These chips can provide support for cell growth, but also, depending on the channel geometry, can serve to manufacture nanomedicines, which will be described in more detail in the next section [15].

### 2.2. Which Technical Considerations Should Be Born in Mind before 3D-Printing Personalized Medicines?

**Figure 2:**
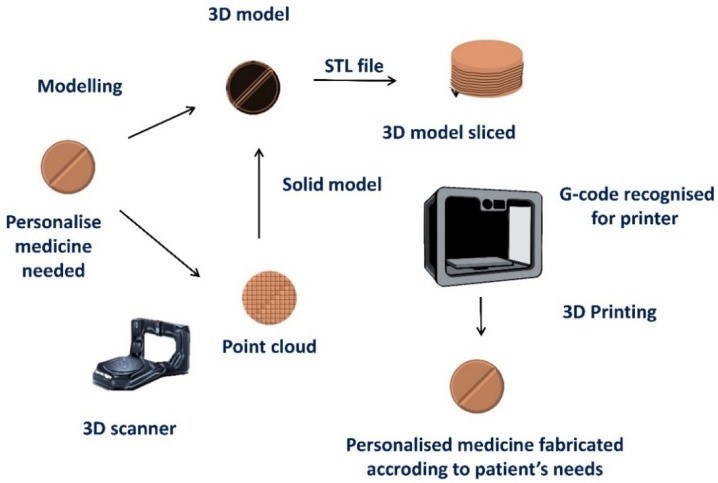
Schematic depicting the steps required for 3D printing of personalized medicines. Modified from: [19].

When manufacturing 3D-printed medicines, it is critical to design the 3D structure using a CAD (computer-aided design) software or obtain a geometry using a 3D scanner (Figure 2) towards creating an stl. file. This file will be sliced into different layers defined by their set height that will be superimposed on top of the other during printing to develop the geometry. The slicing process is carried out with the slicing software integrated into each printer or free-cost available software, such as the Ultimaker CURA software. After the slicing process, a g-code file is generated that can be sent to the printer, dictating the exact coordinates where the “pharmaceutical ink” will be deposited (Figure 2) [19].

There are many 3D printing techniques, but only some of them allow us to manufacture pharmaceutical inks with the required drug loading and/or quality characteristics. In the case of FDM, a flexible filament is required if solid dosage forms are needed to be printed, which needs to be extrudable and loaded with the required stable quantity of the drug [20]. Upon entering the extrusion head of the printer, the filament melts or more accurately becomes malleable, and the molten material can be extruded and deposited layer by layer according to the structure that has been designed. The stability of the drug throughout this process is paramount to ensure that, progressively, the layers are superimposed until the 3D structure of the solid dosage form is generated.

Stereolithography (SLA) requires a photopolymerisable liquid resin that can be mixed with our active ingredient in the form of a solution or suspension, which, when exposed to ultraviolet light, will solidify. Controlling the positions in which the laser beam irradiates the resin will determine the overall geometry of the structure [21]. Other techniques, such as SSE, also known as pressure-assisted extrusion of semisolid material (PAM), in which prefilled syringes are used with a semiviscous mixture of active ingredients and excipients that are extruded using a pneumatic or piston system and deposited on the printer platform according to the indicated coordinates in the G-code, are also commonly employed [22].

### 2.3. What Are the Great Challenges to Bringing This Technology to Clinical Practice?

The length of printing is critical in the production of an adequate number of printed units. Considering oral solid dosage forms, this could range from 7 s to 15 min [23]. However, this is still far lower compared with compression in a conventionally industrial tablet press able to produce millions of tablets per hour. 

The cost of printing needs also be considered as the price, for example, of 3D-printed solid dosage forms is difficult to lower than that of generic compressed tablets. Even though the cheapest FDM printers cost around EUR 100–200, the printing of medicines necessitates that the medicines meet quality attributes and an adequate decontamination protocol is in place to ensure the absence of cross contamination between batches of printed medicines, which is challenging for FDM printers of the lower price range. To the best of our knowledge, there is only one 3D printer for medicines that operates under good manufacturing practice (GMP), M3DIMAKER, currently commercialized at EUR ~80,000 [23]. To overcome the cross-contamination issues, FabRx has implemented printing within blister packaging. 

Pharmaceutical-grade excipients are required with clear audit trails for the manufacture of medicines or drug-loaded filaments, which limits the choice of available techniques utilized for manufacture. Regulatory authorities and ethics committees also have limited experience in handling these products, and available pharmacopoeia tests, such as disintegration, might prove challenging for 3D-printed solid dosage forms. Finally, training health professionals in 3D printing technologies, even if 3D printers are increasingly becoming a household item, requires experience, knowledge, and training to ensure that the printed medicines meet the quality standards. Suitable training should be implemented in points-of-care to ensure that the clinical translation of this technology takes place.

### 2.4. What Are the Differences between Conventional Drug Manufacturing and 3D Printing?

**Figure 3:**
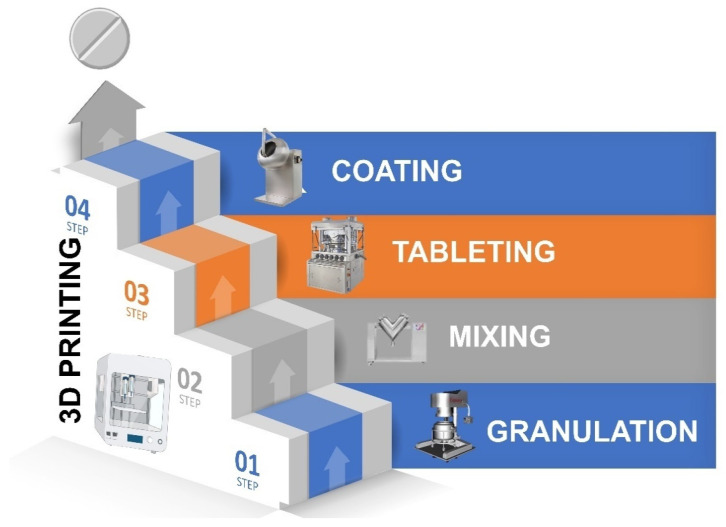
Comparison of conventional solid dosage form manufacturing vs. 3D printing.

Three techniques are mainly used in the pharmaceutical industry to manufacture medicines, direct compression, wet granulation, and dry granulation. The manufacturing process of solid dosage forms, such as tablets, requires a series of sequential steps and can be a complex process. Direct compression is the simplest method since the API is simply mixed with the excipients and the mixture is transferred to the tableting machine, and after compression, a coating of the solid pharmaceutical form may be required. However, sometimes it is not possible to perform direct compression directly because the powdery material does not compress well and a granulation process has to be carried out beforehand. The granulation process allows us to bind the drug with other excipients, forming granules that exhibit much better flow and compaction properties. To granulate, the binder can be added in the presence of water or ethanol. which is called wet granulation, or using compaction rollers, dry granulation. After the granulation process, granules are mixed with other excipients, such as disintegrants or lubricants, followed by tableting. A coating layer may be required in an additional step (Figure 3).

However, through 3D printing, a single step is required once the “pharmaceutical ink” is ready [24]. Depending on what type of medicine we want to print, the type of 3D printing technique should be carefully selected. In Table 1, the main advantages and disadvantages for each technique are described to understand which one meets the requirement according to the type of API. In the following section, the manufacturing of pharmaceutical ink will be described in more detail to understand which type of 3D printing method fits our requirements. 

### 2.5. How the Pharmaceutical Ink Can Be Manufactured for 3D Printing?

**Figure 4:**
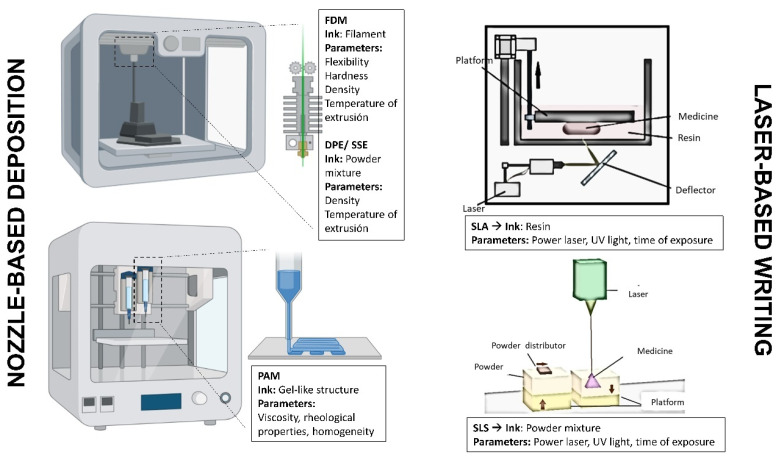
Types of 3D printing techniques commonly used in the manufacture of personalized medicines. Key: fuse deposition modelling, FDM; direct powder extrusion, DPE; semisolid extrusion, SSE; pressure-assisted microsyringes, PAM; stereolithography, SLA; selective laser sintering, SLS.

The two main groups of 3D printing techniques commonly used for manufacturing personalized medicines in clinical points-of-care are nozzle-based deposition systems, also known as material extrusion techniques, and laser-writing systems, also known as vat photopolymerisation (Figure 4). Nozzle-based deposition systems include FDM and its alternatives, direct powder extrusion (DPE), SSE, and PAM. Laser-based writing includes stereolithography (SLA) and selective laser sintering (SLS). The type of ink and critical parameters to control differ from each other [25,26,27,28,29]. 

For FDM, the pharmaceutical ink consists of a filament that contains the drug and the excipients. The filament has to possess adequate characteristics for printing, such as an adequate diameter, flexibility, and hardness. Otherwise, the printer is not able to print. The diameter ranges in most FDM printers between 1.75 and 2.85 mm. Deviations in the average diameter of around 10% are usually accepted, but higher than this, printing is not accurate. The challenge of using this technique is to fabricate optimal filaments [30,31]. 

These filaments are manufactured by a hot extrusion process using an extruder. In this case, a powder mixture of active substances (API) is prepared with the corresponding excipients and incorporated into the extruder inlet hopper. Through the hopper, the powdery mixture is incorporated and mixed by an endless screw, where heating elements are connected that cause a malleable viscous mixture to form. The malleable viscous mixture is forced to leave through a small die, which should be slightly larger than the diameter of the filament required. As the filament comes out of the die, this is pulled and wound into a coil; as it cools, it becomes rigid. During this process, the filament usually suffers from a contraction process, which should be taken into account to match the final filament diameter requirements. 

Even though the process may look simple, several hurdles should be overcome. At the industrial level, the equipment is larger and more versatile, but on a smaller scale, it is more complex. First, controlling the temperature of extrusion is key, as thermolabile drugs could undergo degradation. Commonly, temperatures above 30 °C of the glass transition temperature of the excipient mixture are desirable. If drug degradation occurs, additional excipients with low glass transition temperatures, such as polyethylene glycol, should be included in the mixture to bring down the extrusion temperature. Otherwise, the API can be incorporated at the end of the extrusion process through an additional hopper, limiting the contact time at the extrusion temperature. If excipients melt, the extrusion temperature should not exceed the melting temperature of those as the filament will not cool down fast enough to keep its shape and integrity at the exit from the die. Additionally, the filament has to be flexible, but also have sufficient hardness. Plasticizers play a key role and should be added when necessary. The latter parameters can be measured through texture analyser equipment. However, there is some controversion about which should be the most appropriate tensile properties to ensure successful FDM printing [32,33,34,35].

Density is also a key parameter during FDM to calculate the final dose in our solid dosage formulations. Based on the density and drug loading within the filaments, the final volume of the dosage form should be calculated, and accordingly, the dimensions of the 3D medicine should be designed. Once the filament has been fabricated and the object designed, the medicine should be sliced, and additional key parameters should be chosen before printing, such as layer height (usually 0.1–0.2 mm), which impacts structure resolution and printing time (the lower the layer height, the higher the resolution and the higher the time needed for printing) [36], the temperature of the building platform to ensure good adhesion during the process, and the extrusion temperature of the nozzle printer, which it is commonly 5–10 degrees higher than the temperature needed during hot-melt extrusion. Finally, the printing speed will be adjusted. The slower the printing, the better the resolution, but the process can be very long in time [37]. The most common excipients used in FDM are polylactic acid (PLA), polyvinyl alcohol (PVA), Soluplus, ethylcellulose (EC), Eudragit, hydroxymethyl cellulose (HMPC), hydroxypropyl cellulose (HPC), and polycaprolactone (PCL) [38].

One of the main advantages of this technique is the formation of amorphous solid dispersions that enhance the solubility of poorly water-soluble drugs and, hence, oral bioavailability [39]. Additionally, the temperature is high enough to limit the risk of microbiological contamination, and the water content is limited, enhancing long-term drug stability. The hardness of 3D-printed FDM solid dosage forms tends to be very high, which may result in poor disintegration and drug release [20]. However, the main challenge to overcome when using FDM is the manufacturing of high-quality filaments and working with thermolabile APIs. The solution for the first issue is to use direct power extrusion (DPE) or semisolid extrusion (SSE) instead of FDM, and for the letter challenge, PAM techniques can be used instead [40,41,42,43,44,45].

DPE or SSE are alternative techniques that have emerged to overcome the difficulties to implement FDM in clinical settings. In this case, the nozzle has a metallic chamber connected that allows the direct incorporation of the powder mixture (the pharmaceutical ink) rather than the prefabrication of a filament. Similar to FDM, the powder mixture containing the API and the excipients should be heated ideally 30 °C above the glass transition of the mixture and allow for equilibration to ensure a good heating transfer amongst all powder particles before printing. Otherwise, the 3D-printed medicine will exhibit heterogeneous amorphous domains that can affect the dissolution profile and oral bioavailability. Additionally, poor resolution can be obtained if the powder mixture is not heated evenly. Several examples of personalized medicines, such as paediatric formulations, have been demonstrated using this technique [41,42,43,44].

PAM has been extensively used in tissue engineering as a high temperature is not required for printing, and hence, it is not a limiting factor for cell viability. However, this technique has also been employed in the manufacture of personalized medicines, such as tablets [22], oral bucodispersable films [46,47], and parenteral implants [48].

Bioinks can generally be described as a formulation of cells that is suitable to be processed by an automated biofabrication technology [49]. Aqueous formulations of polymers or hydrogel precursors that contain biological factors would be considered biomaterial inks, which would become bioinks following the addition of cells into that formulation [49]. As a rule of thumb, these biomaterial inks should be biocompatible and biodegradable, and additionally for implants, they have to be sterilized and permeable. When printing with bioinks, the key factor is their rheological behaviour. Typically, inks are viscous semisolid materials. The bioink should possess a minimum viscosity to be able to maintain its structure after being deposited on the platform but, at the same time, it should not be too rigid structures that do not allow adequate cell growth and oxygen permeability. In the case of solid dosage forms, solvents may be required for printing. This is a hurdle as a postprinting step is necessary to ensure that all solvents are eliminated before administration. This is a risk, especially for those solvents more toxic in which the ppm left in the medicine is extremely low, necessitating quality assurance [49]. In this case, the bioink is placed inside a microsyringe that has a small die to control efficiently the deposition of the semisolid material on the platform. These microsyringes are commonly disposable, which reduces the risk of cross contamination and can ensure sterility every time they are used. PAM printers either have a pneumatic system or are mechanically activated with a piston or rotating screw. In the case of the pneumatic system, it uses compressed air to force the ink out through the nozzle. By pressing on the ink, it flows as cylindrical filaments that can be latticed with UV light, enzymes, chemicals, or heat to generate structures with better mechanical properties. Special attention must be paid to ensure that the pressure exerted does not affect cell viability. The printing speed is usually much lower than with FDM printers to achieve better resolution [50,51,52]. The most common excipients used in PAM are Carbopol, polyethylene glycol (PEG), hydroxypropyl cellulose (HPC), hydroxymethyl cellulose (HPMC), and polyvinylpyrrolidone (PVP) [38].

The laser-based writing technologies applied to the fabrication of personalized medicines can be divided into two main techniques, stereolithography (SLA) and selective laser sintering (SLS). SLA is one of the techniques that have a higher resolution compared with other 3D printing techniques, reaching up to 10–25 microns. In this case, the pharmaceutical ink is a mixture of a light-curing resin together with our API. The general printing concept consists of a bath with resin composed of monomers and a photoinitiator capable of being activated when interacting with ultraviolet light. When the UV light irradiates the photoinitiator, free radicals are released, being capable of interacting with other monomers forming rigid polymer chains. When this happens, the resin that was previously in a liquid state hardens and forms a hard plastic solid material. During this process, the API is retained within the rigid polymer structure.

There are two techniques of 3D printing with stereolithography, top–down or bottom–up. If the printing is from top to bottom, the UV light beam is located above the resin tank, so the platform inside the tank will move down as the solid layers are formed. At the beginning of the process, the platform is submerged in the surface at a distance equivalent to the desired thickness with the first layer so that the UV light falls on the surface, forming the first layer of solid. Only in those points where UV light strikes, the resin solidification occurs. As the UV light beam is very small, good resolution can be achieved with this technique. When the first layer is already printed, the platform lowers another distance equivalent to the thickness required by the next layer, covering the solid layer formed with more liquid resin that will be the next layer to be polymerized. Using the other bottom–up printing technique, the UV light beam would be located under the resin tank, which would have a transparent window that would allow light to pass through. In this case, the platform goes up as the layers are formed, and the platform is progressively raised according to the dimensions of each layer to be printed [19,30,53]. 

When using this SLA printing technique, it is necessary to make a postprinting process consisting of washing the printed product with usually isopropyl alcohol to remove excess resin, followed by a curing process using UV light to harden the structure and finish the polymerization process. This step is essential since the polymer formed no longer contains free radicals, which are found in the excess liquid resin and have a proven genotoxic nature [54,55,56]. Although free radicals are mostly eliminated after the postprinting process, their administration for human use is limited, being necessary to develop biocompatible resins for their administration inside the body, for example, in the case of tablets or implants. This technique, however, is widely used in the field of dentistry [57]. 

In the SLS technique, the pharmaceutical ink consists of a layer of powder containing the API along with the necessary excipients onto which the laser light beam will fall directly. The powdery mixture is placed on a platform that is heated slightly below the melting point of the powder. Due to the action of laser light, the powder undergoes a sintering process in which the particles fuse and solidify [58]. The unmelted powder serves as a support for the geometric shape during printing, so unlike the SLA and FDM techniques, there is no need to add any support. Once the first layer has been printed, the platform lowers the height corresponding to a layer that usually ranges between 50 and 200 microns. There is a roller that spreads the powdery mixture after every layer so that the process is homogeneous. This process is repeated until the printing process of all layers is complete. Once the printing is finished, the printing chamber must be cooled, and then the printed structure can be removed and left to rest for a while until it acquires the optimal mechanical properties and the part does not deform. The postprinting process consists of removing excess dust that may remain adhered to our structure. The main problem associated with this technique is the excess powder generated, API with the excipients, which is usually expensive [59]. 

In both laser-based writing techniques, the most critical parameters that should be adjusted before printing are the composition of the pharmaceutical ink, the laser power, and the time of exposition from each layer. If the laser power is too strong, it may degrade sensitive drugs to UV light, but very low radiation can result in poor medicine resolution as the resin is not properly cured or the powder is not bonded appropriately.

Before printing a medicine, it is key to bear in mind the main characteristics of the API, for example, if it is thermolabile or it can degrade easily under UV light. In those cases, PAM can offer an alternative in which neither temperature nor UV light is needed during the process. Additionally, we need to consider which is the target product profile of our medicine. Sustained-release tablets can be easily manufactured using FDM or DPE techniques, while immediate-release tablets are more challenging, taking into account the high mechanical strength obtained with the latter techniques. SLS can be a good alternative to those. Even though PEDGA has emerged as a less toxic resin for SLA, still, its safety for oral administration has not been demonstrated specially in chronic therapies, and hence, SLA has been relegated to other uses that require high precision, such as manufacturing of microfluidic chips or dentistry applications. 

## 3. Implementation of 3D Printing in Personalized Solid, Topical, Parenteral Dosage Forms and Medical Devices

### 3.1. Solid Dosage Forms

Powder binder jetting is the only 3D printing technology that has reached the market at the industrial level. Aprecia laboratories have implemented this technology to manufacture Spritam, a 1000 mg levetiracetam oral dispersible tablet. It does not require heat to fabricate the tablet; it just relies on a powder bed and a liquid binder that makes each layer stick to the other. The tablets are directly printed in the blisters, minimizing the need to harvest dosage forms or recirculate unprinted powder. The preformed orodispersible tablet shell and lid comes out directly using an automated zip dose assembly system. The disintegration time is below 1 min, facilitating deglutition and fast onset of action [60].

**Figure 5:**
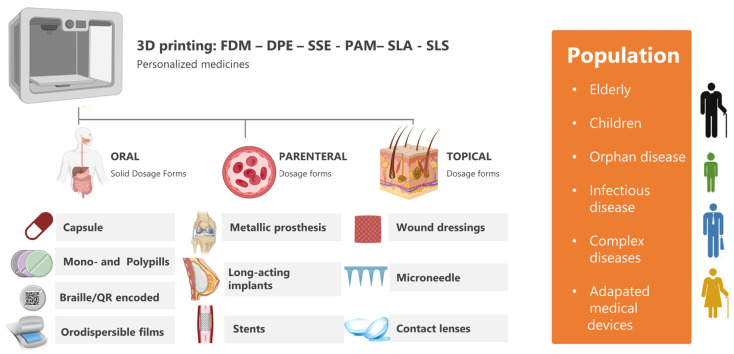
Application of 3D printing technologies to manufacture personalized medicines.

However, nozzle-based extrusion systems and laser-writing systems are more commonly used in research, and these technologies are moving faster to clinical practice, especially for the manufacture of solid, topical, and parenteral medicines (Figure 5). 

**Figure 6:**
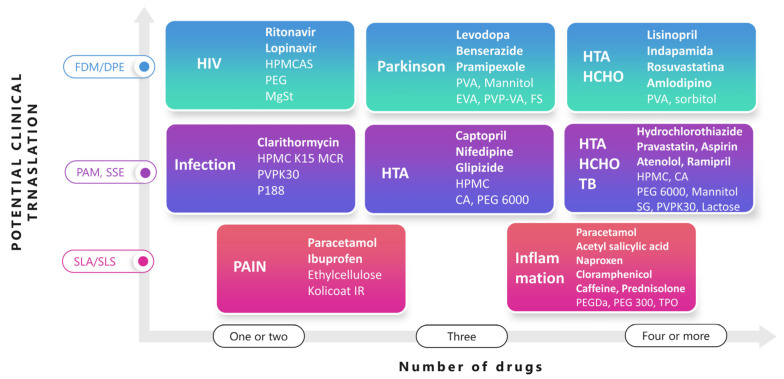
3D printing of mono- and polypills. Key: SSE, semisolid extrusion also known as PAM; FDM, fuse deposition modelling; DPE, direct powder extrusion; SLA, stereolithography; SLS, selective laser sintering; HTA, hypertension; HCHO, hypercholesterolemia; TB, thrombosis; HPMCAS, hydroxypropyl methylcellulose acetate succinate; MgSt, magnesium stearate; PEG, polyethylene glycol; HPMC, hydroxypropyl methylcellulose; CA, acetate cellulose; PVA, polyvinyl alcohol; PEGDA, polyethylene glycol diacrylate; PVPK30, polyvinylpyrrolidone K30; SG, sodium starch glycolate; TPO, thermoplastic polyolefin used as photoinitiator; EVA, ethylene-vinyl acetate copolymer (82:18, *w*:*w*); PVP-VA, vinylpyrrolidone-vinyl acetate copolymer 60:40; FS, fumed silica; P188, poloxamer 188.

Polypills have emerged as a new personalized solid dosage form trying to combine all the medicines that a patient needs in a single tablet, adjusting the dose and the release profile. Commonly, drugs have been combined to treat metabolic syndrome consisting of hypercholesterolemia, hypertension, and hyperglycemia, but also, combined treatments for infectious diseases and pain are gaining attention. Amongst all 3D printing techniques used to manufacture mono- or polypills, FDM is currently the top technology. The main reason is the easier implementation in clinical practice, taking into account that postprocessing is not required as there are no solvents involved in the process, and printed solid dosage forms have suitable characteristics in terms of tensile strength and the ease of modification of the drug release profile. Using FDM technology, polypills containing two, three, four, or even more drugs have been successfully printed. Excipients used can be of a high technical grade similar to the one used currently in the pharma industry to manufacture pills. FDM technology has shown the higher feasibility for implementing 3D-printed polypills in clinical practice followed by PAM and SSE. Additionally, two, three, four, or even more drugs have been successfully printed using this technique, but the postprinting step to remove the solvents utilized during the process is a major challenge to overcome. Polypills have also been printed using SLA and SLS techniques. However, the implementation of these techniques in clinical practice for the fabrication of polypills is more complex, considering the difficulty of incorporating different APIs, the major risk of cross contamination during the process, and the lack of biocompatibility studies with the resins utilized for SLA. In Figure 6, the potential for the clinical translation of different printing technologies is schematically illustrated along with several examples of polypills. 

An easier application of FDM is to print small tablets in size with different APIs each and combine them within the same capsule to achieve a polypill. As an example, a paediatric treatment for HIV has been developed combining minitablets of ritonavir and lopinavir [41]. Due to the low thermal stability of the drugs, DPE was preferred over FDM, making it possible to reduce the extrusion temperature to 80 °C. Hydroxypropyl methylcellulose acetate succinate (HPMCAS) combined with a plasticizer (PEG 4000) and a lubricant (magnesium stearate) was utilised to create a sustained zero-order drug release matrix over a 24 h period (Figure 6). 

Polypills for Parkinson’s disease have been also manufactured using hot-melt extrusion coupled with FDM containing three drugs, levodopa in combination with benserazide, a dopa decarboxylase inhibitor, and pramipexole, a dopamine agonist [61]. Two different composition filaments were fabricated for a rapid-release one containing pramipexole, polyvinyl alcohol, mannitol as a plasticizer, and fume silica as a glidant, and a sustained-release one made of levodopa and benserazide, ethylene-vinyl acetate copolymer (82:18, *w*:*w*), 15% vinylpyrrolidone-vinyl acetate copolymer 60:40 (PVP-VA), and 0.5% fume silica. The tablets were designed with a hollow cylinder inside to make them float in the stomach for over 24 h to ensure a successful absorption of levodopa, considering that it is absorbed in the upper gastrointestinal tract. 

However, polypills manufactured with FDM extrusion can contain four or even more APIS. For example, lisinopril and amlodipine for hypertension, indapamide as a diuretic, and rosuvastatin for dyslipidemia were combined within the same solid dosage form. Filaments were prepared by hot-melt extrusion using PVA and sorbitol as excipients using distilled water as a temporary coplasticizer to reduce the extrusion temperature from 170 to 90 °C [62]. 

The potential for clinical translation of pressure-assisted microsyringes also known as semisolid extrusion has been widely explored. The main limitation is to ensure full solvent removal in the postprocessing step, which increases the manufacturing time and puts at risk patients’ lives. Mono- and polypills have been successfully printed for many different applications, for example, infection, including clarithromycin combined with hydroxypropyl methylcellulose (HPMC), polyvinylpyrrolidone K30 (PVPK30), and poloxamer 188, to form a gel matrix type that floats in the stomach for over 8 h [63]. 

A three-drug polypill has been designed and printed for patients with type 2 diabetes and high blood pressure containing captopril, an angiotensin-converting enzyme (ACE); glipizide, a hypoglycemic drug; and nifedipine, a calcium antagonist [64]. Captopril exhibited a zero-order sustained release due to the incorporation of mannitol as an osmotic agent, which is useful for controlling blood pressure levels over long periods, while glipizide and nifedipine were embedded in a hydrophilic matrix of hydroxypropyl methylcellulose, allowing first-order release by diffusion. Both compartments were physically separated with a layer made of croscarmellose sodium and sodium starch glycolate as disintegrants, PVPK30 as a binder, and mannitol as a diluent.

Additionally, using a four-nozzle PAM printer, a five-in-one-dose combination polypill was successfully printed [22]. The tablet was printed in two different layers, the immediate-release layer containing aspirin as an antiplatelet and hydrochlorothiazide as a diuretic combined with sodium starch glycolate and polyvinylpyrrolidone K30 as a disintegrant and binder, respectively, and a sustained-release layer with atenolol as a beta-blocker, ramipril as an ACE inhibitor, and pravastatin as a 3-hydroxy-3-methylglutaryl–coenzyme physically separated from the other layer by a hydrophobic cellulose acetate shell acting as a permeable barrier along with mannitol as a filler and polyethylene glycol (PEG) as a plasticizer. 

Using laser-writing techniques, such as SLA and SLS, the manufacture of polypills has also been feasible. For pain, SLS has shown the feasibility of printing dual 1 mm in diameter minitablets containing paracetamol and ibuprofen and Kollicoat IR and ethylcellulose as excipients to control drug release for over 24 h [65]. SLA is also a suitable technique for printing polypills. However, the main drawback is the inherent toxicity of the polymerisable resins used. A six-drug tablet was printed containing paracetamol, an antipyretic and analgesic; caffeine; aspirin; naproxen, a nonsteroidal anti-inflammatory; chloramphenicol, a broad-spectrum antibiotic; and prednisolone, an anti-inflammatory corticosteroid [66]. Polyethylene glycol diacrylate (PEGDA) was chosen as a photopolymerisable monomer, and thermoplastic polyolefin as a photoinitiator. To allow the polypill printing, drugs were dissolved individually in PEGDA, thermoplastic polyolefin was used as a photoinitiator (TPO), and PEG 300 Da was poured into the resin tray sequentially. 

Apart from tablets, 3D printing has shown the capability to produce capsule shells either with similar performance to commercially available hard gelatin capsules or able to modify the release of the filling content. Mostly, capsule shells are made of poly(vinyl) alcohol (PVA) and PVA blends with other excipients, such as HPMC [67]. Additionally, the capsule shell can be divided into different compartments, allowing a progressive drug release [68]. 

3D printing techniques have also been demonstrated to be a valid tool for manufacturing oral dispersible films, which can facilitate the manufacturing of those compared with conventional solvent casting techniques especially useful for patients with deglutition problems [69]. SSE has been used to print layer-by-layer benzydamine-hydrochloride-loaded orodispersible films consisting of maltodextrin, a plasticizer such as sorbitol, and a thickening agent, such as hydroxyethyl cellulose [70].

Finally, 3D printing technologies for solid dosage forms have gone one step further to ensure product authenticity by printing QR codes and data matrices on the surface of the tablets to allow track and trace measurement control [71]. Additionally, Braille and Moon patterns can be printed on the surface of the tablets, enabling easier tablet identification for visually impaired patients [72].

Several clinical studies are ongoing to show the real clinical translation of 3D-printed solid dosage forms in patients. Patient acceptability of 3D-printed medicines has been evaluated. The torus shape is preferred over spherical geometries, as well as smaller-size tablets. The colour of the tablet was also a driving parameter for tablet picking [73]. The perceptions of healthcare professionals have also been evaluated, and it has shown that more than 60% of the interviewed healthcare professionals were willing to prescribe 3D-printed medicines, understanding the potential of this new tool in clinical practice [74]. Additionally, 3D-printed tablets have shown great potential to manufacture medicines not commercially available for orphan diseases. 3D-printed isoleucine formulations are used for the treatment of maple syrup urine diseases, a rare metabolic disorder with a worldwide prevalence of 1 in every 185,000 children. Current therapy for children diagnosed with this rare disease consists of full restriction of leucine and supplementation with isoleucine and valine. However, medicines containing isoleucine have to be prepared as an extemporaneous compounding formulation in which the dose needs to be titrated according to the patient’s needs, such as age, weight, and blood levels of these amino acids. When comparing the isoleucine blood levels in patients receiving either isoleucine in conventional capsules or chewable 3D-printed formulations, the latter showed lower blood level variability and higher patient acceptability [75]. This is a promising result to keep investigating the potential of 3D printing in the manufacturing of personalised solid dosage forms.

Apart from Aprecia laboratories, pharmaceutical companies are also investing in 3D printing technologies to manufacture solid dosage forms. Triastek, a Chinese-based pharma company, has one on-going clinical trial authorised by the FDA (Food Drug and Administration) to 3D-print a colon-targeted oral new drug for ulcerative colitis to help improve the safety of the dosage form by enhancing controlled-drug release in specific segments in the colon [76].

These are a few examples of the applicability of 3D printing to oral treatments. However, most of the ongoing clinical trials are focused on the development of personalised prostheses and medical devices [77]. An insight on the advances achieved in this field will be described in the next section.

### 3.2. 3D-Printed Medical Devices

3D printing technologies have also shown great potential in the manufacturing of medical devices. One of the critical parameters is the material chosen for printing, such as the biocompatibility, mechanical properties, and capacity for sterilization. One of the areas that are gaining more attraction in this field is the printing of metallic biomaterials using iron, magnesium, zinc, titanium, cobalt, and stainless steel. Apart from the FDM and SLS, selective laser melting and electron beam melting also are widely used for printing this type of device [78]. Iron is characterized by its high fracture strength, ductility, and hardness. Additionally, it is nontoxic and biodegradable, but the degradation rate of pure iron is very slow, and hence, alloys with Mn and Pd make the degradation rate faster and more uniform. Iron-based alloys can be used as temporary cardiovascular stents [79].

Stainless steel is produced by a combination of iron, carbon, and a minimum of 11% chromium, which has good mechanical properties stronger than bone, heat resistance, and biocompatibility. However, it can cause stress shielding when used as orthopaedic implants, corrosion can occur when in contact with body fluids for longer periods, and inflammation can be the result of the release of certain metals, such as chromium. The combination with other metals makes the alloys more resistant to corrosion and more biocompatible, being used for orthopaedic implants well adjusted to patients’ anatomy, artificial heart valves, needles, catheters, and many other applications [80]. 

Titanium can be used in its pure form or in alloys or composites with ceramics. The main advantage is its high biocompatibility and mechanical strength similar to human bone; however, it is chemically reactive with atmospheric gases, and titanium-based biomaterials require surface treatment. It is one of the most biocompatible metals being widely used in orthopaedics and dentistry [81].

Magnesium is also used for metallic implants as its mechanical properties are comparable to those of human bone, but it is lightweight. However, it degrades too fast, and high rates of corrosion usually take place. It has been used for orthopaedic screws [82]. Zinc has been also used in 3D printing applications for wound closure devices, orthopaedic devices, and cardiovascular stents, exhibiting good corrosion resistance but poor mechanical strength. As an inorganic material, zinc promotes bone tissue growth and suppresses bone tissue loss, making it a good candidate for orthopaedic applications [83]. Cobalt alloys have excellent magnetic properties, wear resistance, and long-term stability. They are the second most used metal in medicine for the manufacturing of implantable devices, such as cardiac pacemakers, defibrillators, hip implants, and coronary stents [78]. 

### 3.3. 3D-Printed Implants

Apart from metals, long-lasting implants and stents can be also manufactured using biodegradable polymers, such as polylactic acid (PLA), polyglycolic acid (PGA), polyvinyl alcohol (PVA), polycaprolactone (PCL), and their copolymers with a degradation time in the body up to 36 months, much longer than natural polymers, such as chitosan, collagen, alginate, and gelatine. A wide range of APIs, such as hormones, cytostatics, anaesthetics, and antimicrobials, have been successfully delivered by FDM 3D printing [84,85,86,87]. 3D-printed rod-shaped implants consisting of PVA and PLA fabricated with FDM slowed down the release, which lasted for up to 300 days [87]. Personalized vaginal rings made of PLA: PCL (8:2 *w*:*w*) and polysorbate 80 loaded with progesterone exhibited a prolonged release for up to 7 days [88]. 3D-printed scaffolds for breast cancer made of PLGA in combination with doxorubicin and cisplatin allowed a sustained release of both cytostatic drugs for over 30 days [89]. Biodegradable stents have been fabricated with a composite of PLA-PCL material printing a PLA core and a PCL shell with a sustained release of over 6 weeks [90]. Stents can also be printed or coated with antimicrobials to prevent infection and anti-inflammatory drugs to reduce postsurgical side effects. For example, amoxicillin and cefotaxime PCL-loaded stents were placed in salivary glands to avoid infection after implantation [91]. 

SLA has also been applied in the manufacture of intravesical bladder devices [92]. One of the main disadvantages of this technique is the release of unpolymerized resin, which can cause acute and chronic toxicity. Novel materials that are more biocompatible should be developed, considering the high resolution that can be obtained using this technique. 3D-printed devices loaded with lidocaine to treat interstitial cystitis and bladder pain were designed to be inserted and removed from the bladder through a urethral catheter. An elastic resin made of a thermoplastic polymer was used, which facilitated the insertion of the device [92]. 

Moving beyond the state of the art, 4D printing is also gaining attention as a new concept to develop patient-centred medicines. 4D printing is defined as the fabrication of dynamic 3D-printed structures that can change their morphology and/or characteristics as a function of time. They are also called smart materials as they suffer from a transformation after certain stimuli, such as a change in pH, temperature, humidity, light, or the presence of a magnetic field. The transformation can be shape-shifting abilities, commonly folding, expansion, shrinkage, and stiffness. For example, 3D-printed scaffolds with NIR-triggered doxorubicin delivery were developed to be implanted immediately after breast surgery. The incorporation of polydopamine provided responsiveness to NIR irradiation. Only under NIR irradiation, the core underwent a sol/gel transition, which resulted in drug release [8]. 

### 3.4. Semisolid and Locally Applied Drugs

3D printing has shown great potential in the manufacture of semisolid and locally applied drugs especially on the skin and on the eye surface. Wound dressings and microneedles are two of the main applications of 3D printing on the skin. Wound healing is a complex process requiring a good equilibrium between cell proliferation, such as angiogenesis and re-ephitelisation, and healing rather than inflammation and scarring [93,94]. PAM is the most suitable technology for manufacturing wound-healing dressings. Novel bioinks are under development made of gelatin methacryloyl (GelMA) and xanthan gum with excellent printability and swelling properties [95]. Chitosan is one of the most promising natural-derived polysaccharides used as a bioink because of its attractive properties, such as biodegradability, biocompatibility, low cost, and nonimmunogenicity [96]. Chitosan–pectin hydrogels with lidocaine have shown promising results, allowing the exudates to be absorbed while maintaining a moist wound healing pain-free environment [97]. Wound dressings loaded with metals, such as copper, zinc, or silver, have been fabricated by hot-melt extrusion using PCL exhibiting excellent antibacterial properties [98]. 

3D-printed microneedles have been manufactured by SLA as a high resolution is key for this type of device. The needle geometry ranges from 25 to 200 µm in height with a 50–250 µm diameter in the base and a 1–25 µm diameter in the tip [99]. 3D-printed solid microneedle arrays have been fabricated using commercial resins coated with cisplatin for skin tumours [100]. Insulin delivery has been also achieved with this type of device. Solid microneedles were printed, followed by a coating process by inkjet printing. The insulin ternary structure was maintained, keeping the performance and eliciting a pharmacological effect equivalent to the subcutaneously administered insulin [101]. Hollow microneedles have been fabricated using Class I resin (Dental SG) in which the microreservoirs were loaded with 360 µL rifampicin solution to treat infection [102]. Similarly, the microreservoirs can be filled with cells previously encapsulated in alginate capsules cross-linked with CaCl_2_, keeping the cell viability [103].

3D printing can be also applied in the manufacturing of contact lenses, opening many new possibilities to explore [104]. 3D-printed lenses should possess certain characteristics to make them suitable for this administration route, such as transparency, sterility, flexibility, permeability to oxygen, and good patient acceptability, amongst others. Several attempts have been made to print contact lenses, but still, they are far from ideal. Contact lenses have been printed using digital light processing and dental commercially available transparent resins. To achieve the desired level of optical transmittance, a postprinting process was required consisting of dip-coating the lenses, followed by a curing process and washing with isopropyl alcohol. Surface roughness remains a challenge [105]. Medicated contact lenses with timolol maleate for glaucoma were printed using FDM, and a biocompatible medical-grade polymer, ethylene-vinyl acetate copolymer–polylactic acid blends. Lenses were printed with a middle aperture to ensure proper vision, considering the lack of optical transmittance of the lens. The surface roughness was improved using this technique, and the drug release was controlled for over 3 days [104]. 

## 4. Implementation of 3D Printing in Personalised Biopharmaceuticals 

Most research in 3D printing has been performed with small molecules. However, the market of biopharmaceuticals products is growing exponentially, and the application of 3D printing in this field, especially PAM, is opening up new ways to manufacture reliable organ-on-chip when incorporating this type of compound. Currently, peptides and proteins have been mostly utilized in 3D printing tissue engineering, mainly cartilage and bone restoration. When bioprinting cell-based scaffolds, the combination with biopharmaceuticals, such as TGF-beta 1 binding peptide, bone morphogenetic protein 2 (BMP-2)–derived peptides, and mussel-derived bioactive peptides, has shown an enhancement of the cartilage and bone growth [106,107,108,109,110]. Bilayered porous scaffolds with GelMA hydrogels as a matrix have been constructed, including an upper layer with bioactive peptides that can adsorb TGF-beta-1 for cartilage repair and a lower layer with hydroxyapatite for subchondral regeneration [107]. PLA scaffolds with enhanced osteogenesis have been developed by coating them with BMP-2-derived peptides conjugated with dopamine. The scaffold promoted the expression of osteogenesis-related genes, such as alkaline phosphatase, osteocalcin, and osteopontin [109]. These are just a few examples of the potential of incorporating peptides and proteins in 3D cultures to mimic closely in vivo tissues using PAM. 

In this case, hydrogels are constructed, and hence, there is no need for heating during printing or postprocessing steps as water is the only vehicle. However, the rheological properties of the hydrogel remain a challenge, and hence, the PAM technique is commonly combined with FDM printing to construct scaffolds to provide enough mechanical strength made of biocompatible polymers, such as PLA, PCL, or PLGA [111]. Peptide hydrogel design focuses on the modulation of the amphiphilic balance of the backbone sequence with an optimal arrangement of hydrophobic–hydrophilic units, allowing for spontaneous physical gelation. The interactions are mainly consisting of H-bonding, charge–charge interactions, and π–π stacking which is triggered by the native protein folding, resulting in different secondary structures, such as α-helices, β-sheet, and hairpin motifs [112,113].

3D-printed solid dosage forms containing biopharmaceuticals are currently under development. A tablet containing alkaline phosphate to treat ulcerative colitis has been fabricated with an ileo-colonic release profile to reduce degradation in harsh media of the stomach [114]. To avoid enzyme degradation during printing, powder bed printing was employed utilizing HPC as a binder. After printing, a coating layer with PEG was included to achieve ileo-colonic release. 

Additionally, polymeric microneedle patches printed by SLA to increase resolution were loaded with insulin to enhance its transdermal delivery. The length of the microneedles was 1 mm with a 1 mm base diameter. A biocompatible Class I resin (Dental SG) was used to minimize the risk of toxicity. A postprinting step was required in isopropyl alcohol under UV radiation at 40 °C to remove any unpolimerised resin left. After the postprinting, an insulin solution containing trehalose, mannitol, and xylitol was inkjet-printed on the microneedles. Once the solvent was evaporated, uniform solid thin films were formed on the needles. Insulin showed a full immediate release for over 30 min after the application on porcine skin [101]. 

3D-printed multiunit implants have been successfully manufactured using polycaprolactone, lauric acid, and melanin as a matrix to enable remote light-controlled protein drug delivery in a spatiotemporal manner. Implants were loaded with insulin in each unit. Upon irradiation with near-infrared light (NIR), heat was generated from melanin, which melted the polycaprolactone and lauric acid matrix, resulting in the insulin release. This implant showed promising results in an vivo model of a diabetic mouse. An efficient decrease of the glycaemia was achieved for over multiple days [115]. 

These results show the potential of 3D printing applied to biopharmaceuticals. Proteins can easily be denaturalized, but peptides can resist greater temperatures, being good candidates for material extrusion techniques, such as FDM or DPE.

## 5. Implementation of 3D Printing in Personalised Nanomedicines

According to the European Commission, nanomaterial refers to “a natural, incidental or manufactured material containing particles, in an unbound state or as an aggregate and wherein one or more external dimensions is in the size range 1 nm–100 nm for 50% or more of the particles” [116]. Nanomaterials can be applied in nanomedicine for medical purposes in three different areas: diagnosis (nanodiagnosis), controlled drug delivery (nanotherapy), and regenerative medicine [117]. Their small size confers their unique properties in medicine due to the high specific surface area in relation to the volume, which leads to a large particle surface energy and, hence, reactivity.

**Figure 7:**
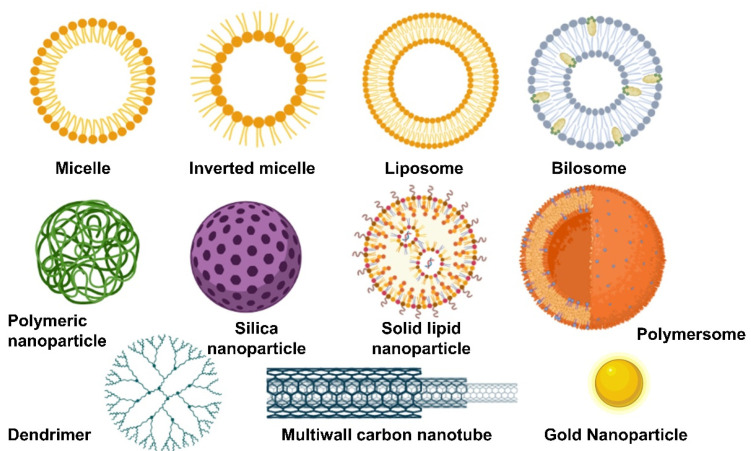
Types of organic and inorganic nanomedicines.

There are a large number of nanomedicines that can be divided into inorganic and organic nanomedicines, such as micelles, liposomes, nanoparticles, nanofibers, and carbon nanotubes, amongst others (Figure 7) [118,119,120,121,122]. Inorganic nanoparticles play a key role in the diagnostics, existing several products commercially available, such as Nanocoll, NanoHSA based on albumin nanoparticles tracked with technetium for nuclear gammagraphy, and SPIONS (superparamagnetic iron oxide nanoparticles), used in magnetic resonance imaging [123]. Several organic nanomedicines are also commercially available, most of them liposomes for cancer and infectious diseases, such as Doxil (pegylated liposomes of doxorubicin), DepoCyte (liposomes of cytarabine), Onivyde (liposomes of irinotecan), and AmBisome (liposomes of amphotericin B for fungal diseases), but also albumin nanoparticles loaded with paclitaxel (Abraxane) and polymeric micelles loaded with paclitaxel (Apealea). 

Apart from liposomes characterized by having a rigid bilayer with cholesterol with prolonged circulation in the blood, transferosomes are greater vesicles for topical drug delivery due to their flexible membrane when cholesterol is replaced by edge activators, such as sodium deoxycholate [124,125]. The flexible membrane allows them to squeeze through pores amongst the stratum corneum, reaching deeper regions of the skin. 

Self-nanoemulsifying drug delivery systems (SNEDDS) are also promising vehicles able to solubilise high quantities of poorly soluble drugs and deliver them across the skin or the gastrointestinal track [126,127,128]. SNEDDS are anhydrous homogeneous liquid mixtures, consisting of oil, surfactant, drug, and/or cosolvents, which spontaneously form transparent nanoemulsion usually between 20 and 200 nm droplet size upon aqueous dilution with gentle agitation [129]. When the physicochemical stability of liquid SNEDDS is poor, they can be adsorbed onto silica nanoparticles and transformed into solid nanomedicines [130,131,132]. 

**Figure 8:**
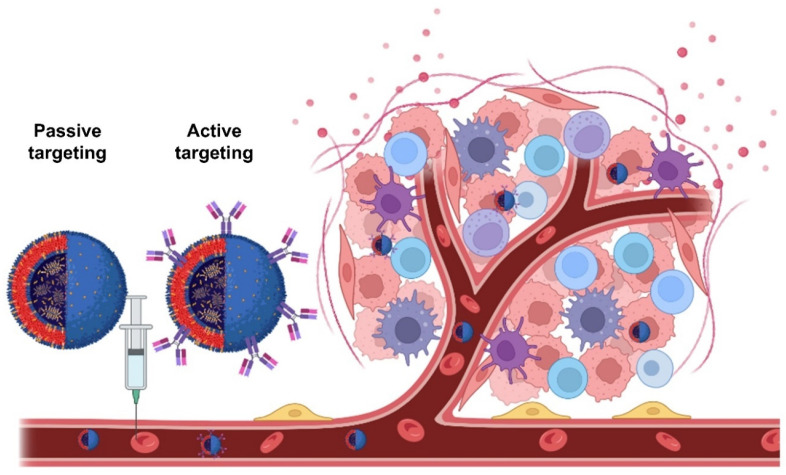
Comparison of passively versus actively targeted nanomedicines.

Polymeric nanoparticles are also promising drug delivery systems currently under research [133,134]. For example, a wide number of publications highlight the potential of PLGA nanoparticles to target tumours using a passive or active approach. Passively targeted nanoparticles make use of biological mechanisms to achieve specific organs or sites of disease, such as phagocytosis by the cells of the reticuloendothelial system (RES) [135] or enhanced permeation and retention (EPR) effect observed in tumours with leaky vasculature, while actively targeted nanoparticles have attached to their surface a targeting moiety, making them selectively interact with a receptor to elicit their effect, which is especially useful to cross the blood–brain barrier [136,137] (Figure 8). The concept of active and passive targeting can be applied to mostly all types of nanomedicines. 

### 5.1. 3D Printed Nanomedicines

3D printing technologies have been used in the manufacturing of nanomedicines. However, nanoparticle concentration is a key factor, taking into account that particle aggregates behave as defects and weaken the 3D-printed structure. In general, achieving high drug-loading nanoparticles in a polymer matrix is challenging due to nanoparticle attractions and Van der Waals–induced aggregations. To improve particles’ homogeneity in liquid suspension, a preprocessing step may be required, such as an ultrasound application, the addition of surfactants, ball milling, and so on [138]. 

Redispersible 3D-printed solid dosage forms containing polyphenols (curcumin and resveratrol) were loaded in polymeric PCL nanocapsules. The latter were embedded in a carboxymethyl cellulose 3D-printed hydrogel by PAM. The polyphenols were partially released over an 8 h period, but not all the active ingredients were released from the nanocapsules, being still a challenge to overcome [139]. Curcumin loaded in liposomes was printed in 3D-printed tissue scaffolds. Curcumin possesses strong antioxidant, anticancer, and osteogenic properties, but it is poorly available due to its lipophilicity. The incorporation of loaded liposomes in 3D-printed calcium phosphate scaffolds provided significant cytotoxicity toward osteosarcoma, whereas it promoted osteoblast viability [140].

3D-printed solid lipid dosage forms consisting of a 3D-printed dissolvable polymer scaffold were fabricated with PLA and PVA with different compartments to load in a second-step solid lipid formulation within a single dosage form. The emulsions were prepared using Gelucire 44/14, Gelucire 48/16, and Kolliphor P188 containing either fenofibrate or clofazimine, lumefantrine, and halofantrine as model drugs. The mixture was prepared under heat and stirring and was loaded into the compartments of the solid dosage form. Once the temperature reached room temperature, the solid lipid system solidified. Different release profiles were achieved depending on the lipid ratio to manufacture the solid lipid systems [141].

Solid SNEDDS were successfully printed directly as a tablet. A semisolid paste was prepared by the fusion method containing dapagliflozin, capryol 90, poloxamer 188, PEG 6000 and 400, and cremophor EL. The lipid system consists of a liquid phase containing oils and cosurfactants and a solid phase with a solid matrix with a surfactant. Once all excipients and drugs were fully melted, they were transferred to a PAM cartridge for 3D printing. The dapagliflozin-loaded SNEDDS 3D-printed tablet showed an immediate release profile (>75% in 20 min) [142]. A similar approach was used to fabricate 3D-printed lidocaine-loaded SNEDDS suppositories to treat hemorrhoids by PAM [143].

### 5.2. Conventional Batch-to-Batch Approach versus Continuous Manufacturing Using Microfluidics Chips

There are multiple conventional technologies utilized at the laboratory scale in the fabrication of nanomedicines. The solvent evaporation–precipitation technique (bottom–up approach) is probably the most widely used [144,145,146]. The scale-up of this technique is very challenging at the industrial level, taking into account the high amounts of remaining solvents that have to be removed and the volume of the tank used to fabricate the particles as well as the impeller speed, and the liquid media can alter significantly the final properties of the particles [147]. Additionally, it is challenging to control the polymorphism and solid state of the drug, crystalline or amorphous, that will determine the physicochemical performance of the drug [144,148]. Spray drying and spray coating can facilitate the scale-up, but controlling the particle size remains challenging [149,150,151,152,153]. 

The conventional batch manufacturing method still does not have any alternatives in the pharmaceutical industry, but the continuous manufacturing approach is gaining attention focused on a process in which raw materials are continually injected into a manufacturing facility, and products are continuously discharged during the operation of the manufacturing processes [154]. Continuous manufacturing requires a fully automated system and continuous monitoring in real time. The incorporation of process analytical technologies on, in, and at the production line along with the implementation of chemometric models facilitates the successful manufacturing of nanomedicine within the specification limits set [155]. 

The investment in the manufacturing of nanomedicines at the industrial level is limited due to (1) lack of infrastructure and in-house expertise, (2) low speed of fabrication compared with the production of capsules and tablets (1 million/h), (3) insufficient batch-to-batch reproducibility requiring a rigorous control of the particle size, and (4) lack of chemical and physical stability [156,157]. The current cost of development and market authorization of nanomedicines is much higher than conventional medicines and results in economically unaffordable products for public healthcare systems and patients [158]. In Europe, the commercialisation of nanotherapeutics is driven by start-ups and small- and medium-sized enterprises [158]. Thus, there is a clinical need for bridging the gap between the development of excellent health solutions and efficient production and development. 

Microfluidic devices are microscale fluidic circuits utilized to manipulate liquid at the nanolitre scale. Fine control of process parameters afforded by microfluidics allows unprecedented optimization of nanomedicine quality and encapsulation efficiency [159]. Automation improves the reproducibility that lacks using conventional technologies, such as solvent evaporation, to produce nanomedicines. In addition, the continuous nature of the microfluidic process is inherently scalable, allowing optimization at low volumes, which is advantageous with scarce or costly material [160]. However, the engineering of microfluidic devices is complex, and there have been some barriers to commercializing these devices that traditional fabrication methods, such as injection moulding using polydimethylsiloxane (PDMS), have failed to address, for example, nonstandard user interfaces, complex control systems, and high cost. However, these barriers may be overcome by 3D printing, which is a more cost-effective technology that has shown a great improvement in terms of channel resolution, and there are a variety of commercialised materials ready to use with ideal properties, such as being transparent, nonfluorescent, and biocompatible [161]. The use of PDMS chips for the manufacturing of nanomedicines has been extensively reviewed previously [162].

However, the use of 3D printing to fabricate microfluidic devices capable of high-throughput synthesis of nanomedicines with tuneable dimensions is feasible [163,164,165,166,167,168]. Utilising a high-resolution 3D printing process based on FDM or stereolithography, reliable patterning of channel features with dimensions of ~200 µm has been demonstrated, resulting in the production of nanomedicines (<100 nm at a production rate of 4 mg/min) [169]. This can be achieved with a single device due to the engineering of flow-focusing microchannels with high aspect ratios, together with the seamless fabrication of high-pressure fluidic ports for world-to-chip interfacing that supports large volumetric flow rates and high-throughput nanoparticle synthesis [170]. 

3D printing microfluidic devices are capable of high-throughput synthesis of nanomedicines with tuneable dimensions, resulting in an enormous advantage compared with the conventional batch method [163,164,165,166,167,171,172]. Still, solvent and unencapsulated drug removal remain a challenge for continuous manufacturing using these devices. The feasibility of 3D-printed microfluidic chips for the manufacturing of nanomedicines has recently been demonstrated, and a few examples are illustrated [15,173]. Nifedipine-loaded polymeric nanoparticles were engineered using 1000 µm in diameter channelled 3D-printed microfluidic chips made of either commercially available resin and SLA or cyclin copolymer olefin and FDM. Particles exhibited a similar drug loading and particle size as those obtained by conventional solvent evaporation [15]. Curcumin loaded in liposomes was manufactured using FDM-printed chips with channels of 1000 µm in diameter. Liposomes with about 200 nm in size and 99% encapsulation efficiency were obtained [174]. Glycyrrhetinic-acid-loaded ethanolic liposomes were successfully prepared by FDM-printed T-shaped microfluidic chips with round channels with 600 µm. Liposomes were 200 nm in size with a 63% drug encapsulation efficiency [173]. Sucrose decorated liposomes loaded with berberine for breast cancer were successfully manufactured in an effective passive micromixing with a “zigzag” bas-relief (Z-chip) and “split and recombine” channels (C-chip) [175]. Better encapsulation efficiencies and lower particle size could be achieved with a smaller channel diameter and more advanced channel geometry. 

## 6. Conclusions and Future Perspectives

The applications of 3D printing in personalized medicine are unimaginable. Medicines can be customized and adapted to patients’ needs. Solid dosage forms can be manufactured by combining one or more drugs within the same solid dosage form to improve patient compliance, facilitate deglutition, or fabricate a new tablet when no medication is available. Regarding parenteral solid dosage forms and medical devices, 3D printing has opened a new range of possibilities, such as implants and prostheses perfectly adapted to the patient’s anatomy, such as metallic implants and cardiovascular stents. Semisolid locally applied drugs also benefit from the personalization provided by 3D printing, for example, to manufacture wound dressings adapted to the wound characteristic of the patient, microneedles to transdermal delivering the drug to deeper regions of the skin, or by printing medicated contact lenses with a sustained release profile to treat certain diseases, such as glaucoma. 

The integration of nanotechnology-based drugs along with 3D printing (“nanoprinting”) brings printed personalized nanomedicines within the most innovative perspectives for the coming years [176,177,178], enabling continuous manufacturing through the use of microfluidic chips, facilitating their translation into clinical practice. Finally, the combination of 3D printing with biopharmaceuticals opens multiple windows to improve current therapies and develop further 3D cultures used in tissue engineering. 

However, the reality is that the clinical translation of 3D printing is very slow. Very few clinical trials are going on regarding the use of 3D-printed tablets to treat a specific pathology for which there is no commercially available medicine. The main reasons behind this fact are the complexity of using 3D printers at high-quality standards, the lack of clear regulatory guidelines, the lack of trained personnel, and the lack of accessibility to good manufacturing practice (GMP) printers that avoid cross contamination. The potential of 3D printing for different medical needs has been widely demonstrated. However, most of the published research has been performed with standard 3D printers that do not comply with the regulatory requirements. For example, there is no assurance that cross contamination cannot occur between the manufacturing of different dosage forms. This is a clear problem when using FDM printers in which the different combinations pass through the same extrusion nozzle, which can be challenging to clean. There are very few printers that can operate under GMP conditions, but the prices at the moment are extremely high, which limits their affordability to implement in clinical settings. Additionally, healthcare personnel should receive suitable training to fabricate personalized medicines with high-quality standards. For that, it is key to ensure knowledge transference from bench to clinical settings and optimization of protocols to minimize variability batch-to-batch. Finally, the main regulatory authorities, such as the Food Drug Administration (FDA), the European Medicines Agency (EMA), and the Pharmaceuticals and Medical Devices Agency (PMDA), should join together to draft a clear regulatory guideline to fabricate 3D-printed medicines directly in clinical settings. The currently published drafts are unspecific with voids that should be amended to guide healthcare personnel and researchers. After COVID, a conformity assessment procedure for 3D printing and 3D-printed products used in a medical context was issued and has paved the way for implementing 3D printing in clinical practice. However, this applies mainly to 3D-printed medical devices rather than medicines. Nevertheless, it is expected that in the next decade, 3D printing changes the path of personalized medicines.

## Figures and Tables

**Table 1:** Comparison of 3D printing techniques utilised for the fabrication of personalised medicines. FDM, fuse deposition modelling; DPE, direct powder extrusion; SSE, semisolid extrusion; PAM, pressure-assisted microsyringes; SLA, stereolithography; SLS, selective laser sintering; Tg, glass transition temperature.

Printing Technique	Type	Key Parameters	Advantages	Challenges	Type of Medicines
Nozzle-based deposition	FDM	Temperature of extrusion Layer height Speed of printing Filament composition and diameter Tg composite	High mechanical strength Availability of pharmaceutical-grade excipients	A suitable filament is required for printingHigh temperatures are usually necessary Thermolabile drugs	Solid dosage forms (easier to obtain sustained-release tablets rather than immediate-release ones) Parenteral implants
	DPE	Temperature of extrusion Layer height Speed of printing Powder mixture	High mechanical strength Availability of pharmaceutical-grade excipients No need for filament prefabrication	High temperature of extrusion Lack of homogeneity during the process Thermolabile drugs	Solid dosage forms (easier to obtain sustained-release tablets rather than immediate-release ones) Parenteral implants
	PAM	Viscosity of the material Speed of printing Layer height Composition of the ink	No need for high temperature High cell biocompatibility	Solvent removal in the postprinting step Poor mechanical strength	Tissue engineering Solid dosage forms
Laser-based writing	SLA	Laser power intensity Time of exposure Type of resin UV wavelength	High resolution No need for high temperature	Toxicity of the resin Postprinting step necessary to remove unsolidified resin UV-sensitive drugs	Dentistry Microfluidic chip fabrication
	SLS	Laser power intensity Time of exposure Type of powder mixture	High resolution No need for solvent	Risk of degradation by laser exposure Excessive waste of powder mixture	Solid dosage forms

## Data Availability

No applicable.
